# Ultrasound-Guided Percutaneous Cryoneurolysis for Post-Thoracotomy Pain Syndrome: A Case Report

**DOI:** 10.7759/cureus.32888

**Published:** 2022-12-23

**Authors:** Joshua Perese, Jessica Oswald, Rodney A Gabriel

**Affiliations:** 1 Emergency Medicine, Loma Linda University Medical Center, Loma Linda, USA; 2 Anesthesiology, University of California San Diego, La Jolla, USA

**Keywords:** ultrasound guidance, percutaneous cryoneurolysis, percutaneous cryoablation, cryotherapy, refractory pain, intercostal neuralgia, post-thoracotomy pain syndrome, cryoneurolysis

## Abstract

Post-thoracotomy pain syndrome (PTPS) is a post-operative thoracotomy complication that is difficult to treat. We describe the first-time use of ultrasound-guided percutaneous cryoneurolysis of the intercostal nerves to successfully treat PTPS refractory to conventional medications and interventions. We report a case of a 40-year-old male with two years of severe PTPS sustained after undergoing a thoracotomy. Treatment with intercostal cryoneurolysis resulted in an immediate 75% improvement in pain for six weeks followed by sustained 50% pain relief for eight weeks. This highlights the potential of this intervention as a radiation-free, safe, and efficacious therapy for chronic PTPS.

## Introduction

Post-thoracotomy pain syndrome (PTPS) is recurrent or persistent pain along a thoracotomy incision for at least two months after an inciting surgery or procedure [1]. Its prevalence ranges from 14% to 83% and results in persistent, difficult-to-treat debilitating pain [1,2]. Though the exact pathophysiologic mechanism remains unclear, current literature posits a neuropathic etiology secondary to iatrogenic intercostal nerve injury [2]. The majority of PTPS studies focus on prevention via intraoperative and perioperative multimodal analgesia including medications with adjunctive regional nerve and fascial plane blocks [1,2]. The existing literature on treatment for developed PTSP is limited to case reports and studies with small sample sizes of novel treatments that include botulinum toxin injection, nerve blocks, nerve ablations, and neuromodulations [1-4].

Cryoneurolysis is an efficacious interventional analgesic modality that has been used perioperatively to prevent the development of post-thoracotomy pain [5]. Cryoneurolysis involves the freezing of target nerves to temperatures between -20oC and -100oC to achieve reversible axonal degeneration; this is in contrast to cryoablation, in which the goal is permanent tissue destruction [6]. Recently, a small number of case reports have described real-time image-guided cryoneurolysis as an effective therapy for PTPS; however, the literature is limited to case reports that describe a computed tomography (CT) guided approach. Ultrasound (US) has the added benefit of being more ubiquitously available and the ability to visualize the dynamic pleura in real time during inhalation and exhalation [7,8]. In 2006, there was one published case report describing US-guided cryoneurolysis as a successful treatment for subacute nociceptive post-operative thoracotomy-related incisional pain that was present one month after surgery. While technically not meeting the definition of PTPS with only month of post-operative pain, this case is the most recent contribution to the literature related to US-guided cryotherapy for established PTPS, and it highlights the potential use of US to provide cryoneurolysis to the intercostal nerves [7,9,10]. To our knowledge, there are no prior publications describing US-guided intercostal cryoneurolysis as a treatment for developed PTPS. We describe the first case where US-guided cryotherapy of T3, T4, T5, T6, T7, and T8 intercostal nerves resulted in immediate and sustained improvement in pain, function, mood, and mobility in a patient whose pain was present for two years and refractory to medication optimization and multiple other targeted interventions.

This case report was undertaken with written authorization by the patient in compliance with the Health Insurance Portability and Accountability Act (HIPAA) and an abstract of this article was presented at the Annual Regional Anesthesiology and Acute Pain Medicine Meeting on May 13, 2021.

## Case presentation

A 40-year-old male presented for evaluation of chronic left-sided lateral chest wall pain sustained after undergoing a thoracotomy two years prior. He underwent esophagectomy for dysphagia that was complicated by an intrathoracic abscess requiring an open thoracotomy for abscess drainage.

During his initial evaluation in the pain clinic, the patient reported an 8/10 burning pain on the numeric pain rating scale (NPRS) that originated below his scapula and wrapped anteriorly and superiorly into his axilla. His pain was exacerbated with left arm movement and light touch. He noted that it affected his sleep, exercise, ability to work, and quality of life. His pain regimen included daily home physical therapy exercises, weekly acupuncture, and medical therapy including oxycodone 30 mg and gabapentin 3,600 mg daily. His physical examination was significant for allodynia to his elliptical thoracotomy incision and left anterolateral chest wall along the T3, T4, T5, T6, T7, and T8 ribs.

His history and physical examination were consistent with PTPS with associated multi-level intercostal neuralgia. The initial intervention performed was a left serratus anterior plane (SAP) block (30 mL of 0.25% bupivacaine and 10 mg of dexamethasone) with scar trigger point injections resulting in 50-70% improvement in pain for one week before gradually regressing to his baseline pain within three weeks post-procedure. The ephemeral response led to the decision to perform a US-guided percutaneous cryoablation of the intercostal nerves.

Procedure method

**Figure 1:**
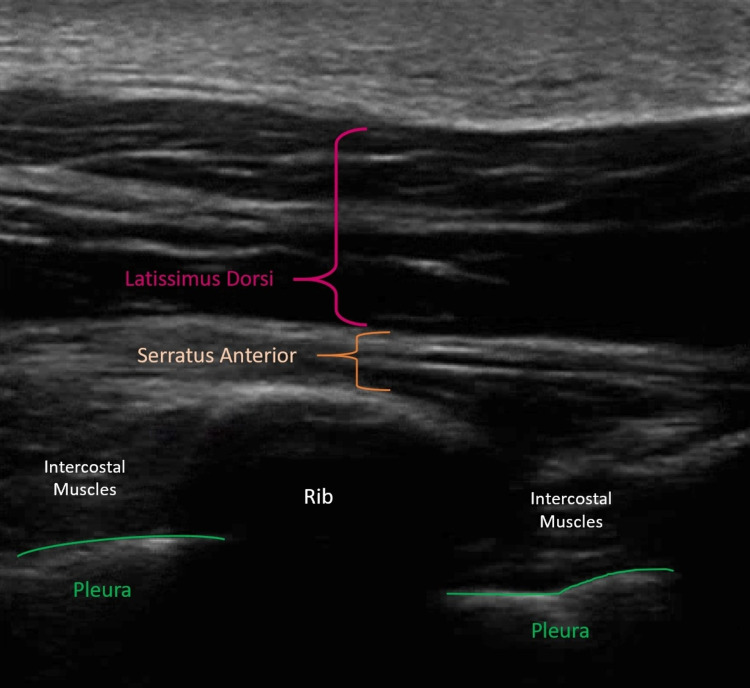
Normal intercostal sonoanatomy in the sagittal plane. From superficial to deep, the pertinent anatomy includes latissimus dorsi, serratus anterior, intercostal muscles, rib(s), and pleura.

**Figure 2:**
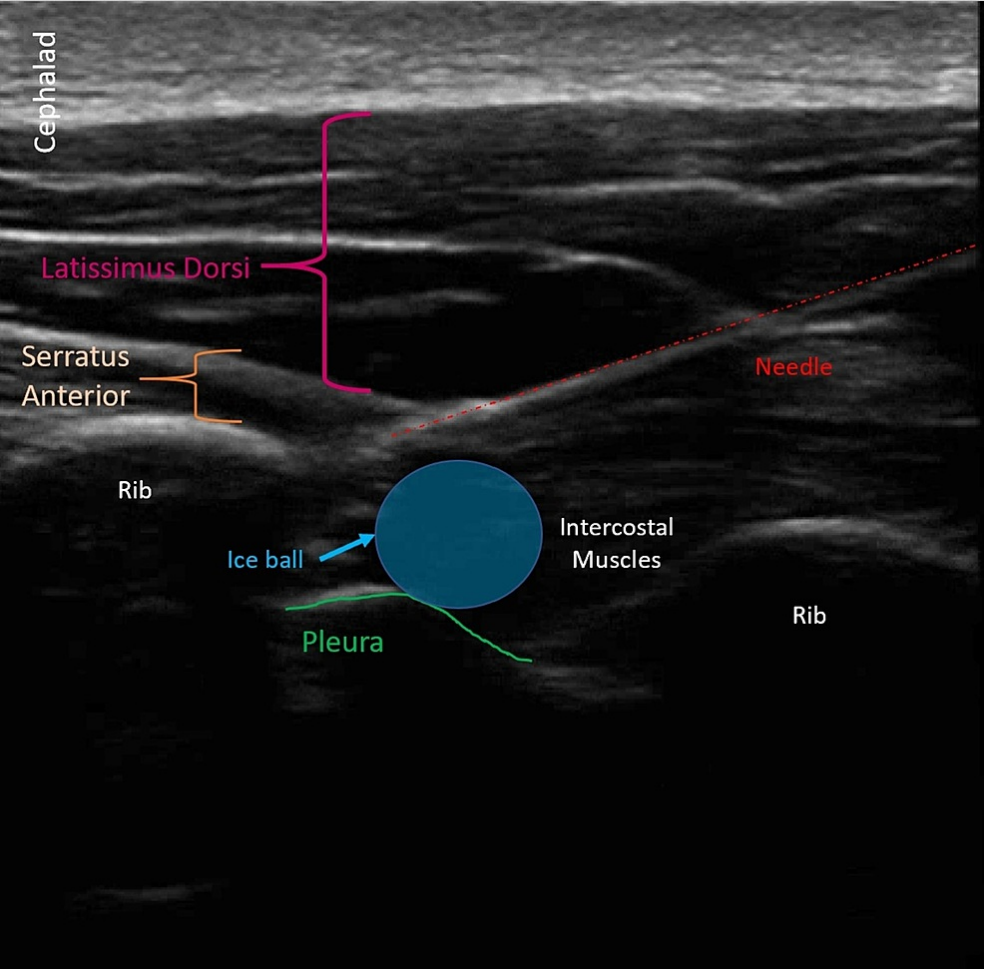
Ultrasound imaging of percutaneous cryoneurolysis of intercostal nerves A still image from the ultrasound-guided percutaneous cryoneurolysis in the sagittal plane. An echogenic needle is seen producing an ice ball at the posterior and inferior aspect of the rib. Relevant sonoanatomy is redemonstrated.

After obtaining informed consent, the patient was positioned prone and remained awake and alert throughout the procedure. Ribs 3-8 were marked, and the patient was draped in a sterile fashion. The skin and soft tissues were anesthetized with 2 cc of lidocaine 1% at each site. Using US guidance, a 12-gauge introducer angiocatheter was inserted 5 cm lateral to the spinous process at the position of the posterior intercostal space (Figure 1) and advanced through intercostal muscles towards the fifth rib (Figures 1, 2). The cryoneurolysis probe (CRYO Pain Blocker™, Epimed, Dallas, TX) was then passed through the introducer and placed posterior to the fifth rib to target the T5 intercostal nerve. Two cycles of 2 minutes of cooling to a temperature of -70°C (nitrous oxide) with 30 seconds of thawing between cycles were performed (Figure 2). The introducer and probe were then removed. Using new introducer angiocatheters, this procedure was repeated to treat the left T3, T4, T6, T7, and T8 intercostal nerves. The patient experienced no hemodynamic or neurologic sequelae.

Results

Within 30 minutes following cryoneuroylsis of the intercostal nerves, the patient reported 75% improvement in his pain (via NPRS) that was sustained for six weeks, followed by a continued 50% reduction in pain for eight weeks for a total of 14 weeks of pain improvement. The patient also reported an overall subjective 80% improvement in mobility, function, mood, and the ability to work longer and start exercising. Notably, his daily oxycodone use was weaned from 30 mg to 20 mg.

## Discussion

PTPS, once developed, is a notoriously difficult-to-treat chronic condition that results in mild-to-severe functional and psychosocial impairments [1,2]. While emphasis is placed on preventing the development of PTPS using perioperative multimodal analgesia, few interventional treatments have been thoroughly investigated for the treatment of developed PTPS.

Perioperative prevention of post-thoracotomy pain has comprised the bulk of PTPS research. While intraoperative epidurals remain the current preventive gold standard, emerging literature highlights paravertebral, intercostal nerve, erector spinae, serratus anterior, and pectoralis I and II blocks as potential epidural adjuncts and/or alternatives to an intra-operative epidural [1,2].

The current literature discussing treatment options for developed PTPS is limited to case reports and small sample sized studies exploring botulinum toxin injection, nerve or plane blocks, neuromodulation, and nerve ablation. A recent case study found botulinum toxin injection in painful sites of PTPS previously refractory to oral medications to provide 80% pain improvement for 12 weeks post-injection; however, this is in contrast to one other case study in which the pain improvement was 50% after treatment [11,12]. Literature for nerve or plane blocks for chronically developed PTPS such as intercostal nerve, paravertebral, erector spinae plane (ESP), and SAP blocks appears promising, with recent ESP and SAP block case reports noting 90-100% immediate pain improvement with 50-100% pain relief at one-month post-procedure follow-up [13,14]. Neuromodulation treatments, such as spinal cord and dorsal root ganglion, have demonstrated significant improvement in chronic PTPS pain, ranging from 70% to 75% pain improvement for one to two years post-procedure; however, given the small sample sizes, the procedures’ invasive nature, and lack of control arms, these also require further research and investigation [15,16]. The current literature on available interventions for developed PTPS highlights the benefits, limitations, and overall need for larger sample sized studies. More invasive modalities (i.e., neuromodulation techniques) appear to provide prolonged pain relief (i.e., one to two years post-procedure); however, the invasive nature of these procedures may limit their utility [15,16]. In contrast, some relatively less invasive modalities, such as US-guided nerve/plane blocks or subcutaneous botulinum toxin injection, appear to lack consistent results in sustained analgesia, with case reports of ESP blocks reporting pain relief for 12 hours to one month and an early case report of subcutaneous botulinum toxin injection reporting a 50% decrease in pain [12,13,17].

Modern cryoneurolysis is a percutaneous technique wherein a metallic probe delivers rapid and expanding pressurized nitrous oxide to freeze a targeted nerve, resulting in Wallerian degeneration and cell death via formation of intracellular ice crystals and disruption of the extracellular osmotic gradient [5,18]. The ensuant conduction blockade and analgesia is a reversible process in which the duration of cryoanalgesia is dependent on the rate of axonal regrowth [3,19]. Cryoneurolysis has been used for numerous intractable pain conditions and offers benefits over thermal ablation through preservation of the epineurium and perineurium, allowing for potential nerve regeneration and decreased risk of neuroma formation [5]. Additionally, it has a wide reported variation in the duration of analgesia ranging from three months to three years, with all prospective studies reporting maximal pain reduction at one month and sustained significant pain reduction after one year, making it a viable long-term treatment option with “as needed” repeat treatments [5].

The first intercostal nerve cryoablation was conducted intraoperatively in 1974 to prevent the development of PTPS [5]. While early studies reported analgesic efficacy, the landmark-guided procedure resulted in inadvertent pneumothorax and injury to the lung leading to its discontinuation [5]. However, with modern advances in real-time image-guidance modalities, cryoablation has resurfaced as a favorable therapy for perioperative post-thoracotomy pain management and a potential therapy for developed PTPS. Notably, despite most randomized controlled trials (RCTs) of intraoperative cryoneurolysis demonstrating no increased risk of post-operative pain, two RCTs reported statistically significant increased incidence of neuropathic pain development at multiple time intervals up to one year post-thoracotomy, with other small studies also noting a potential association [20]. The development of neuropathic pain was possibly related to a double-crush phenomenon in which more than one mode of injury occurs at the same time, such as intraoperative cryoablation performed at the time of thoracotomy [20].

The literature regarding cryoneurolysis for developed PTPS is limited to a few case studies and a small sample sized retrospective analysis, which utilized landmark or CT guidance [9,10]. These studies describe efficacious analgesia of varying duration ranging from eight weeks in one case report to 11 months (median) in a retrospective analysis [9,10]. While other studies utilized CT guidance, our case report found US-guided cryotherapy to be effective in providing significant pain reduction that was immediate and sustained while limiting patient radiation exposure and providing continuous dynamic imaging of the pleura during inspiration and expiration [9,10]. There is no previous study describing US as a means to deliver cryoneurolysis therapy to the intercostal nerves for developed and chronic PTPS refractory to medication optimization and targeted nerve blocks. We report a case in which a single US-guided cryoneurolysis intervention was used in such a patient to provide a safe, radiation-free means of immediate pain relief that was significant and sustained over 14 weeks compared to the four weeks of pain relief reported in studies utilizing US-guided nerve/plane blocks [12-17].

Considering medicine’s evolving focus on patient-centered outcomes and the national goal of decreasing opioid use for chronic pain disorders, there is increasing interest in novel therapies for the prevention and treatment of chronic pain syndromes such as PTPS. While this case report highlights the potential of US-guided cryoneurolysis as a safe and efficacious intervention for developed PTPS in patients who are refractory to other therapies and can be repeated, as necessary, for long-term pain relief, studies with larger sample sizes and RCTs are required to more adequately characterize the safety and efficacy of this technique compared to the current standard of care.

## Conclusions

We described the first case report of US-guided percutaneous cryoneurolysis of the intercostal nerves that successfully provide 14 weeks of significant pain relief in PTPS refractory to conventional medications and interventions.
